# A Review of the Microbial Production of Bioactive Natural Products and Biologics

**DOI:** 10.3389/fmicb.2019.01404

**Published:** 2019-06-20

**Authors:** Janette V. Pham, Mariamawit A. Yilma, Adriana Feliz, Murtadha T. Majid, Nicholas Maffetone, Jorge R. Walker, Eunji Kim, Hyo Je Cho, Jared M. Reynolds, Myoung Chong Song, Sung Ryeol Park, Yeo Joon Yoon

**Affiliations:** ^1^Geisinger Commonwealth School of Medicine, Scranton, PA, United States; ^2^Baruch S. Blumberg Institute, Doylestown, PA, United States; ^3^Department of Chemistry and Nanoscience, Ewha Womans University, Seoul, South Korea; ^4^School of Life Sciences and Biotechnology, Kyungpook National University, Daegu, South Korea; ^5^Natural Products Discovery Institute, Doylestown, PA, United States

**Keywords:** natural products, biologics, biological activity, microbial cell factories, genetic engineering, combinatorial biosynthesis, production improvement

## Abstract

A variety of organisms, such as bacteria, fungi, and plants, produce secondary metabolites, also known as natural products. Natural products have been a prolific source and an inspiration for numerous medical agents with widely divergent chemical structures and biological activities, including antimicrobial, immunosuppressive, anticancer, and anti-inflammatory activities, many of which have been developed as treatments and have potential therapeutic applications for human diseases. Aside from natural products, the recent development of recombinant DNA technology has sparked the development of a wide array of biopharmaceutical products, such as recombinant proteins, offering significant advances in treating a broad spectrum of medical illnesses and conditions. Herein, we will introduce the structures and diverse biological activities of natural products and recombinant proteins that have been exploited as valuable molecules in medicine, agriculture and insect control. In addition, we will explore past and ongoing efforts along with achievements in the development of robust and promising microorganisms as cell factories to produce biologically active molecules. Furthermore, we will review multi-disciplinary and comprehensive engineering approaches directed at improving yields of microbial production of natural products and proteins and generating novel molecules. Throughout this article, we will suggest ways in which microbial-derived biologically active molecular entities and their analogs could continue to inspire the development of new therapeutic agents in academia and industry.

## Introduction

Natural products originate as secondary metabolites from a myriad of sources, including terrestrial plants, animals, marine organisms, microorganisms, terrestrial vertebrates and invertebrates (Chin et al., 2006). These structurally and chemically diverse molecules act as a remarkable class of therapeutics to heal various ailments. The earliest documentation of the application of natural products to improve human health dates back to the ancient Mesopotamia’s sophisticated medicinal system from 2900 to 2600 BCE (Borchardt, 2002; Siddiqui et al., 2014). By the early 1900’s, approximately 80% of all medicines were obtained from plant sources (Sneader, 1997; Siddiqui et al., 2014). The discovery of penicillin from *Penicillium notatum* by Alexander Fleming in 1928 marked a significant shift from plants to microorganisms as a source of natural products (Fleming, 1944). Since then, microorganism-derived compounds have been utilized in medicine, agriculture, food industry and scientific research (Sanchez et al., 2012). The early years of antibiotic research discovered streptomycin from *Streptomyces* *griseus* (Waksman et al., 1946), chloramphenicol from *Streptomyces venezuelae* (Duggar, 1948), chlortetracycline from *Streptomyces aureofaciens* (Ehrlich et al., 1947), cephalosporin C from *Cephalosporium acremonium* (Newton and Abraham, 1955), erythromycin from *Saccharopolyspora erythraea* and vancomycin from *Amycolatopsis orientalis* (Geraci et al., 1956). Given these historical successes, large pharmaceutical companies have continued to invest in this traditional domain (Dias et al., 2012). Currently, approximately 60% of approved small molecule medicines are related to natural products, and 69% of all antibacterial agents originate from natural products (Patridge et al., 2016; Matsumura et al., 2018). However, many natural compounds with potential as novel drug candidates occur in low concentrations in nature, often making drug discovery and development burdensome and economically impractical. Therefore, an emerging alternative solution is to express biosynthetic genes from the original producers in microbial hosts, notably bacteria and fungi (Song et al., 2014). Engineered microbes can produce appreciable amounts of scarce natural compounds, thereby facilitating the synthesis of the target novel compound and potent derivatives, as well as the validation of their activities (Matsumura et al., 2018).

The natural product sector is not the only area that has undergone substantial growth or utilizes therapeutic products generated in/from living organisms. Prokaryotic and eukaryotic microbial cells, in combination with the advancement of recombinant DNA techniques, have been responsible for an explosion of biologics. Biologics are a set of molecules whose active pharmaceutical ingredients are derived from living organisms such as animals, plants, microorganisms, human blood products, and tissue transplants that are too complex to be produced through organic synthesis (Revers and Furczon, 2010). They can be categorized into five main classes: (1) monoclonal antibodies, like trastuzumab (Herceptin^®^) and rituximab (Rituxan^®^); (2) blood factor derivatives, like coagulation factor VIIa (NovoSeven RT^®^) and epoiten alfa (Epogen^®^); (3) vaccines; (4) enzymes; and (5) recombinant proteins, such as immunomodulatory cytokines, and thrombolytic agents (Lacana et al., 2007). Since the approval of recombinant human insulin and recombinant human growth hormone as some of the first modern biopharmaceuticals, large numbers of additional biopharmaceuticals have been developed, approved, and marketed using different microbial expression systems; many more are currently in the development pipeline (Graumann and Premstaller, 2006). After the successful production of the recombinant human insulin Humulin^®^, *Escherichia coli* quickly became the prevalent expression platform in the 1980s when the biopharmaceutical sector emerged and was followed by yeast *Saccharomyces cerevisiae* (Sanchez-Garcia et al., 2016). Microbial cells constitute the majority of hosts employed in the production of currently approved recombinant pharmaceuticals for human treatment, mainly because of their lack of unconventional post-translational modifications, proteolytic instability, poor solubility and activation of cell stress responses (Graumann and Premstaller, 2006). This demonstrates that microbial hosts represent convenient and robust platforms for the efficient production of recombinant proteins despite some bottlenecks and obstacles.

Herein, we will summarize the biological activities and applications of a variety of natural products and biologics and review the microbial systems used to produce these pharmaceutical compounds. We will also cover past and current attempts at improving the microbial production of these biological molecules and generating new molecules using diverse engineering approaches. In addition, we will discuss the challenges of the production of natural products and biologics in microbial systems and advances that can help overcome them for drug discovery and development. Future prospects for cutting-edge developments and technological advances in microbial production of bioactive natural products and recombinant proteins as the most valuable sources of therapeutics are also discussed.

## Biological Activities of Natural Products and Biologics

Natural products have diverse biological activities relevant to human health, including antibiotic, antifungal, anticancer, immunosuppressive, anti-inflammatory, biofilm inhibitory activities, etc. In this section, we will focus on the biological activities of natural products, which can be grouped into several categories. The biological activities of microbial recombinant proteins will be also reviewed.

### Antibiotics

**FIGURE 1:**
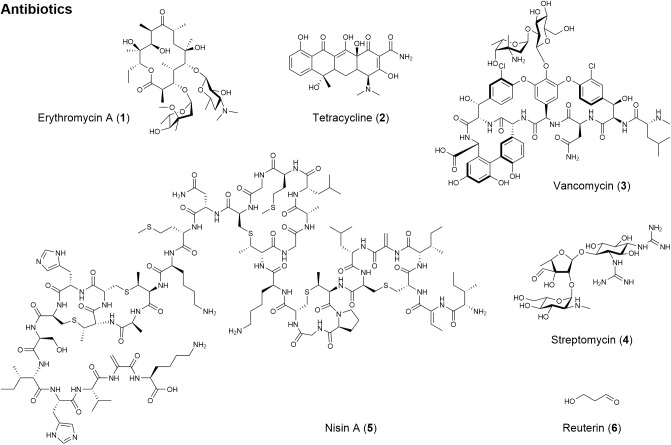
Structures of natural products with antibiotic activity.

Natural products are a rich source for antibiotic drug development, but the most clinically useful of these scaffolds can be classified as polyketides, non-ribosomal peptides, and aminoglycosides (Wright, 2014). Polyketides, assembled by polyketide synthases (PKS), make up one of the largest classes of chemically diverse natural products and are among the most important secondary metabolites for their applications in medicine, agriculture, and industry (Tae et al., 2007). For example, pikromycin was the first known polyketide antibiotic produced from *S. venezuelae* in 1950 (Vazquez, 1967; Jung et al., 2006). It has been reported that pikromycin is very potent against multi-drug resistant respiratory pathogens (Woo et al., 2014). Another remarkable polyketide antibiotic with significant clinical applications is erythromycin A (1; Figure 1 and Table 1), which was first discovered in 1952 as a broad-spectrum antibiotic produced by *S. erythraea* (McGuire et al., 1952). This antibiotic is prescribed to a treat wide variety of bacterial infections, such as respiratory and gastrointestinal infections, whooping cough, syphilis, and acne, especially in patients who have adverse reactions against penicillin (Cobb et al., 2013). While many natural antibiotics fail to inhibit Gram-negative organisms, tetracyclines (2; Figure 1 and Table 1) are active against both Gram-positive and Gram-negative bacteria (Chopra and Roberts, 2001; Demain, 2009).

**Table 1:** Biological activities of microbial-derived natural products and biologics.

Name	Origin	Biological activity	References
**Antibiotic**			
Erythromycin A (1)	*Saccharopolyspora erythraea*	Antibacterial	McGuire et al., 1952; Zhang et al., 2010; Cobb et al., 2013
Tetracycline (2)	*Streptomyces rimosus*	Antibacterial	Chopra and Roberts, 2001; Demain, 2009
Vancomycin (3)	*Amycolatopsis orientalis*	Antibacterial	Geraci et al., 1956; Dasgupta, 2012
Streptomycin (4)	*Streptomyces* *griseus*	Antibacterial	Schatz et al., 1944; Waksman et al., 1946
Nisin A (5)	*Lactococcus lactis*	Antimicrobial	Li and Vederas, 2009; Gyawali and Ibrahim, 2014
Reuterin (6)	*Lactobacillus reuteri*	Antimicrobial	Talarico et al., 1988; Gyawali and Ibrahim, 2014
**Antifungal Agents**			
Amphotericin B (7)	*Streptomyces nodosus*	Antifungal	Abu-Salah, 1996; Tevyashova et al., 2013
Ieodoglucomide C (8)	*Bacillus licheniformis*	Antifungal	Tareq et al., 2015
**Anticancer and Antitumor**			
Bleomycin (9)	*Streptoalloteichus hindustanus*, *Streptomyces verticillus*	Squamous cell carcinomas, Hodgkin’s lymphomas and testis tumors	Einhorn and Donohue, 2002; Demain and Vaishnav, 2011
Ddaunorubicin (10)	*Streptomyces peucetius* and various related strains	Acute lymphoblastic or myeloblastic lymphoma	Di Marco et al., 1981; Giddings and Newman, 2013
**Immunosuppressant/Anti-inflammatory Agents**			
Rapamycin (11)	*Streptomyces rapamycinicus* (formerly, *Streptomyces hygroscopicus* ATCC 29253),* Streptomyces iranensis*, and *Actinoplanes* sp. N902-109	Immunosuppressive, antifungal, antitumor, neuroprotective, neuroregenerative, and lifespan extension activities, growth inhibitory activity against several fungi	Vezina et al., 1975; Mann, 2001; Law, 2005; Pan et al., 2008; Anisimov et al., 2011; Song et al., 2015; Yoo et al., 2017
FK506 (12)	*Streptomyces tsukubaensis* and several *Streptomyces* species	Immunosuppressive, antifungal, anti-inflammatory, neuroprotective and neuroregenerative activities, rheumatoid arthritis treatment	Tanaka et al., 1987; Mann, 2001; Migita and Eguchi, 2003; Demain, 2014; Ban et al., 2016; Yoo et al., 2017
**Biofilm-Inhibitory Agents**			
Cahuitamycins (13)	*Streptomyces gandocaensis*	Inhibitors of *Acinetobacter baumannii* biofilms	Park et al., 2016
**Others**			
Avermectins (14)	*Streptomyces avermitilis*	Onchocerciasis and lymphatic filariasis	Shen, 2015
Mollemycin A 20 (15)	*Streptomyces* sp. (CMB-M0244)	Gram-positive and Gram-negative bacteria, antimalarial activity	Blunt et al., 2016
Lipstatin (16)	*Streptomyces toxytricini*	Pancreatic lipase inhibitor for obesity and diabetes	Weibel et al., 1987; Sanchez et al., 2012


As previously mentioned, penicillin is a well-known antibiotic secondary metabolite from *P. notatum* and is effective against Gram-positive bacteria, which are responsible for diseases such as scarlet fever, pneumonia, gonorrhea, meningitis, and diphtheria (Fleming, 2001; Tan and Tatsumura, 2015). Penicillin belongs to non-ribosomal peptide antibiotics along with vancomycin (Fischbach and Walsh, 2006). Non-ribosomal peptides, assembled by non-ribosomal peptide synthetase (NRPS), possess bioactivity that can be exploited for therapeutic applications and are amongst the most widespread and structurally diverse secondary metabolites. Vancomycin (3; Figure 1 and Table 1) is another potent non-ribosomal peptide against pathogenic bacteria, including *Clostridium difficile*, *Listeria monocytogenes*, *Streptococcus pneumoniae*, *Staphylococcus epidermidis*, and methicillin-resistant *Staphylococcus aureus* (MRSA) (Dasgupta, 2012).

Aminoglycosides are another class of antibiotics that act by binding to the rRNA subunit of the 30S bacterial ribosome and inhibiting protein synthesis (Moazed and Noller, 1987). Streptomycin (4; Figure 1 and Table 1) produced by *S. griseus* is the first aminoglycoside discovered in 1944 and effective against pulmonary tuberculosis (Schatz et al., 1944). Since the discovery of streptomycin, aminoglycoside antibiotics such as kanamycin, gentamicin, sisomicin, and lividomycin have been discovered and widely used to treat infectious organisms that have developed resistance against streptomycin after prolonged use (Park et al., 2013). Despite their excellent antibacterial activity, aminoglycosides have met with resistant organisms. In order to combat antibiotic resistance to aminoglycoside antibiotics, semi-synthetic aminoglycoside antibiotics were specifically tailored to shield against these enzymes (Van Bambeke et al., 2017). Semi-synthetic aminoglycoside antibiotics such as amikacin, netilmicin, dibekacin, and isepamicin are developed as a result of semi-synthetic derivatives of the natural product (Miller et al., 1976; Leggett, 2015).

Natural antimicrobials have also been important to the food industry in terms of food safety against foodborne pathogens. Microbes such as lactic acid bacteria, produce a wide range of chemicals that have been shown to inhibit the growth and development of other microbial species. Nisin A (5; Figure 1 and Table 1), a bacteriocin produced by *Lactococcus lactis*, is approved to preserve food in over 50 countries and is very active against Gram-positive bacteria resistant to conventional antibiotics (Li and Vederas, 2009; Gyawali and Ibrahim, 2014). Reuterin (6; Figure 1 and Table 1) from *Lactobacillus reuteri* has been shown to have antimicrobial activities against foodborne pathogens and spoilage organisms when evaluated in milk, dairy, and meat products (Talarico et al., 1988; Gyawali and Ibrahim, 2014).

### Antifungal Agents

**FIGURE 2:**
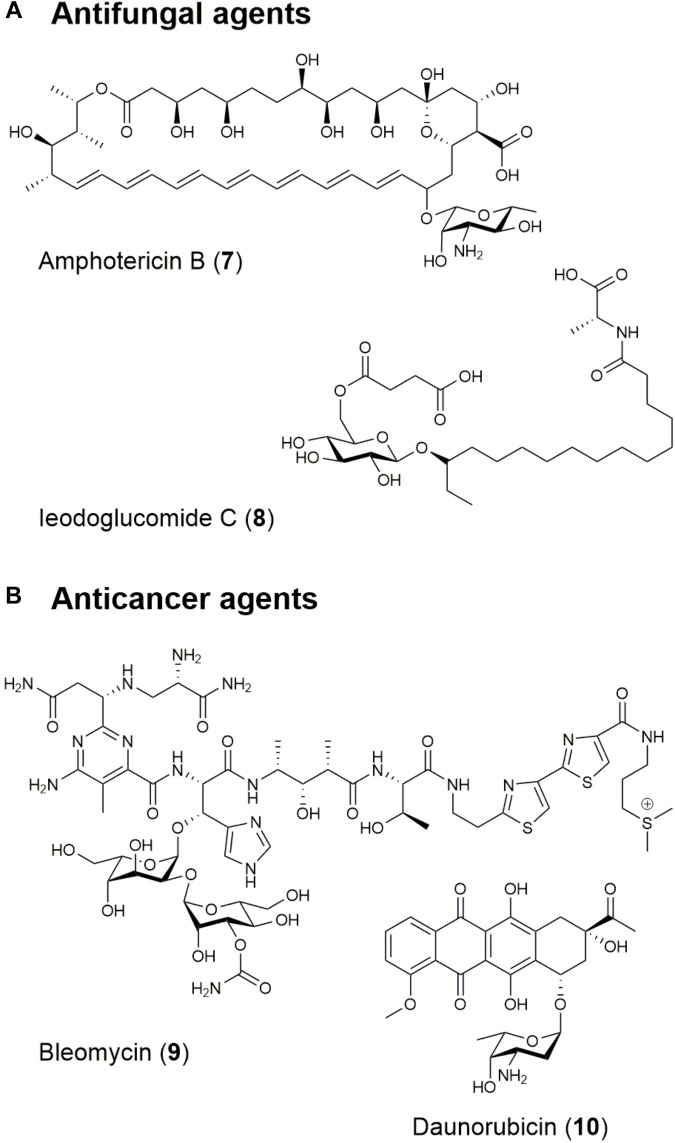
Structures of natural products with **(A)** antifungal and **(B)** anticancer/antitumor activities.

Nystatin, one of the first effective polyene antifungal agent, was obtained from *Streptomyces noursei* in 1950 and effective against *Aspergillus* species (Stanley and English, 1965). Clinically, nystatin plays a significant role as a topical antifungal agent in treating oral, gastro-intestinal, and genital candidosis (Fjærvik and Zotchev, 2005). Amphotericin B (7; Figure 2 and Table 1) is also a traditional polyene antifungal product of *Streptomyces nodosus* utilized against life-threatening fungal infections caused by *Aspergillus* species, and especially effective in patients who have undergone organ transplantation, received aggressive chemotherapy or with acquired immunodeficiency syndrome (Abu-Salah, 1996; Tevyashova et al., 2013).

Recently, in a review of natural products with anti-*Candida albicans* activity, 71 substances of the 142 evaluated were determined to have antifungal activity under the criteria of having minimum inhibitory concentration (MIC) values below 8 mg/mL (Zida et al., 2017). The glycolipids ieodoglucomide C (8; Figure 2 and Table 1) and ieodoglycolipid were isolated from the marine bacterium *Bacillus licheniformis* and exhibited antifungal activities with a 21 μg/L MIC against *Aspergillus niger*, *Rhizoctonia solani*, *Botrytis cinerea*, *Colletotrichum acutatum*, and *C. albicans* (Tareq et al., 2015). Both ieodoglucomide C and ieodoglycolipid also exhibit good antibiotic properties against *S. aureus*, *Bacillus subtilis*, *Bacillus cereus*, *Salmonella typhi*, *E. coli* and *Pseudomonas aeruginosa* with MICs ranging from 0.01 to 0.05 μM, establishing these compounds as strong potential candidates for the development of new fungicides (Tareq et al., 2015).

### Anticancer Agents

There are many microbe-derived anticancer agents that have been evaluated through clinical trials. For instance, the polyketide actinomycin was isolated from *Streptomyces parvulus* in 1940 and was the first antibiotic shown to have anticancer activity (Waksman and Woodruff, 1940; Hollstein, 1974). In particular, actinomycin D, also known as dactinomycin, is approved by FDA and has been widely used in clinical practice as an anticancer drug for treating many tumors, such as Wilms’ tumor, childhood rhabdomyosarcoma, Ewing’s sarcoma, and metastatic, non-seminomatous testicular cancer.

Another notable example is the therapeutic combination of the microbial product bleomycin (9; Figure 2 and Table 1), the plant compound etoposide, and the synthetic agent cisplatin, which has played a significant role in increasing the cure rate for disseminated testicular cancer from 5% in 1974 to 90% in 2011 (Einhorn and Donohue, 2002; Demain and Vaishnav, 2011). Bleomycin is a glycopeptide produced by *Streptoalloteichus hindustanus* and has been used for squamous cell carcinomas, melanomas, sarcomas, testicular, and ovarian cancer, Hodgkin’s and non-Hodgkin’s lymphomas, and testis tumors as an anticancer agent (Demain and Vaishnav, 2011). Its derivative, blenoxane is also used clinically with other compounds against lymphomas, skin carcinomas, and tumors of the head, neck, and testicles (Demain and Vaishnav, 2011). The anthracyclines are also an important family of polyketides produced by *Streptomyces* species by iterative PKS pathways and include daunorubicin (10; Figure 2 and Table 1) (Di Marco et al., 1981), epirubicin (Cersosimo and Hong, 1986), and doxorubicin (Metsä-Ketelä et al., 2008). The FDA approved the use of daunorubicin and doxorubicin for cancer therapy in the 1960s. Daunorubicin is used in the treatment of acute lymphoblastic or myeloblastic lymphoma. Meanwhile, doxorubicin is used in the treatment of breast cancer, solid tumors in children, soft tissue sarcomas, and aggressive lymphomas (Giddings and Newman, 2013).

Among numerous recent examples, rapamycin, wortmannin, and geldanamycin have been found to have antiproliferative activities during clinical use as novel chemotherapeutic agents (da Rocha et al., 2001). Rapamycin, a natural product derived from *Streptomyces rapamycinicus* has anticancer activity in addition to its immunosuppressive, anti-inflammatory, and antifungal activities (Kim et al., 2014). It performs antitumor activity on a tumor cell by hindering its proliferation, triggering apoptosis, and inhibiting angiogenesis (Law, 2005). Wortmannin is a fungal furanosteroid derivative of *Penicillium funiculosum* (Davidson et al., 2013). It has shown as an effective selective inhibitor of phosphoinositide 3-kinases (PI3Ks) and PI3K- related enzymes which are play a key role in intracellular signaling pathways (Sieber et al., 2010). A study on the proliferation and apoptosis of human breast MCF-7 cells treated with wortmannin uncovered that wortmannin shows antitumor activity by triggering apoptosis and impeding proliferation of cancer cells by suppressing PI3K/Akt signaling and NF-κB protein expression (Yun et al., 2012). Geldanamycin is a benzoquinone ansamycin antitumor compound derived from *Streptomyces hygroscopicus* var. *geldanus* (Singh et al., 2010). Geldanamycin prevents ATPase activity by binding to the heat shock protein and hindering the stability and function of oncogenic protein kinases involved in signal amplification cascade that controls proliferation and apoptosis (Singh et al., 2010). Geldanamycin and its analogs play a key role as anticancer agent in multiple myeloma, breast, and prostate cancer (Gorska et al., 2012). Another example is epothilone, an anticancer agent produced from mycobacterium *Sorangium cellulosum*. It obstructs microtubule depolymerization thereby causing G2-M interphase arrest of the cell cycle (Molnar et al., 2000). There are also marine microbial natural products that have anticancer activities, such as dolastatin, which is originated from cyanobacteria of the genera *Symploca* and *Lyngbya* (Simmons et al., 2008).

### Immunosuppressive Agents

**FIGURE 3:**
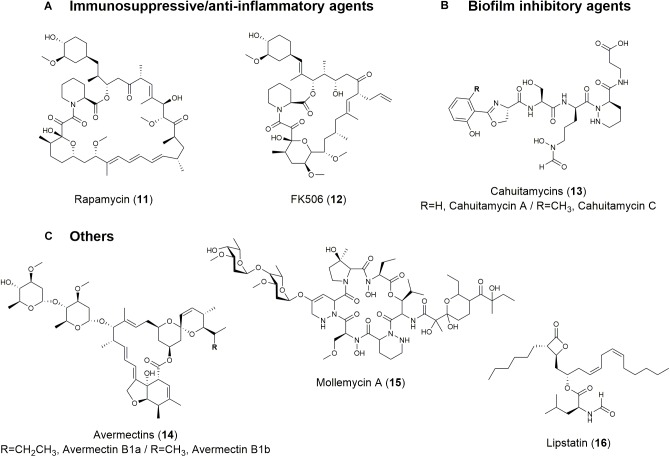
Structures of natural products with **(A)** immunosuppressive/anti-inflammatory, **(B)** biofilm-inhibitory, and **(C)** other activities.

Rapamycin (also known as sirolimus) (11; Figure 3 and Table 1) and FK506 (tacrolimus) (12; Figure 3 and Table 1) are microbial natural products with immunosuppressive properties. Rapamycin blocks the proliferation of most cell types in response to activation by IL-2, IL-3, platelet-derived growth factor, epidermal growth factor, and insulin (Vezina et al., 1975). Rapamycin also exhibits synergism with other immunosuppressants, such as cyclosporin, to significantly reduce kidney toxicity and acute renal allograft rejection (Yoo et al., 2017). This compound has been developed to coat coronary stents and prevent organ transplant rejection and lymphangioleiomyomatosis; it was approved by the FDA for wider use in 1999 (Mann, 2001). In addition to its immunosuppressive activity, rapamycin possesses several other biological activities, including antitumor, neuroprotective/neuroregenerative, antineoplastic, and lifespan extension activities (Law, 2005; Pan et al., 2008; Yoo et al., 2017).

FK506 is also an immunosuppressive drug and was first discovered in soil samples containing *Streptomyces tsukubaensis* and several other *Streptomyces* species (Tanaka et al., 1987). FK506 is used to minimize organ rejection and to induce immunosuppression via calcineurin inhibition and interruption of T cell activation pathway (Migita and Eguchi, 2003). It has been demonstrated to be more effective than cyclosporin and non-toxic in low doses (Demain, 2014). The discovery of its immunosuppressive activity led to its use in heart, liver, and kidney transplants with overwhelming success (Demain, 2014). Like rapamycin, FK506 possesses various biological activities, including antifungal, anti-inflammatory, neuroprotective, and neuroregenerative activities (Ban et al., 2016).

### Anti-inflammatory Agents

Some natural products also have anti-inflammatory activities. FK506 has shown efficacy in the treatment of refractory rheumatoid arthritis, a chronic inflammatory disease (Migita and Eguchi, 2003). Rapamycin also inhibits the inflammatory response after spinal cord injury by diminishing the activation and proliferation of inflammatory cells and the expression of inflammatory cytokines, thereby reducing secondary injury in the spinal cord and providing a neuroprotective effect (Song et al., 2015). Recently, strepsesquitriol, isolated from *Streptomyces* sp. SCSIO 10355, has been found to have anti-inflammatory activity through the inhibition of tumor necrosis factor-α production in lipopolysaccharide-activated macrophages (Yang et al., 2013). Salinamides A and B from marine *Streptomyces* sp. CNB-091 also displayed potent topical anti-inflammatory activity through a phorbol ester-induced mouse ear edema assay (Trischman et al., 1994). One study evaluated 7 peptides found in the *Faecalibacterium prausnitzii* supernatant, all belonging to a protein named microbial anti-inflammatory molecule (Breyner et al., 2017). These peptides were able to inhibit the NF-κB pathway *in vitro* and showed anti-inflammatory properties *in vivo* in a dinitrobenzene sulfate-induced colitis model (Breyner et al., 2017).

### Biofilm Inhibitory Agents

Parasitic microorganisms adhere to solid surfaces and form layers of a complex polysaccharide matrix called a biofilm that confers resistance against antibiotics as wells as inflicts significant chronic bacterial infections (Singh et al., 2017). Analogs of 5-benzylidene-4-oxazolidinones are small molecules derived from marine natural products. These molecules inhibit 89% of biofilm formed by MRSA at 0.78 μM and disperses pre-formed biofilms at 4.7 μM (Edwards et al., 2017). A synthetic library of 2-aminoimidazole triazoles was able to successfully inhibit 94% of biofilm formation in *Acinetobacter baumannii* and MRSA at 100 μM (Rabin et al., 2015). Another recent example is cahuitamycins A-C (13; Figure 3 and Table 1) derived from the marine bacterium *Streptomyces gandocaensis*. Cahuitamycins have been evaluated as inhibitors of *A. baumannii* biofilms and it has been found that cahuitamycin C shows half maximal inhibitory concentration (IC_50_) at 14.5 μM. Modifications of cahuitamycins through selective mutasynthesis have yielded cahuitamycins D and E with an increased the potency of antibiofilm activity against *A. baumannii* (Park et al., 2016). The FDA-approved antitumor agent actinomycin D has also significant biofilm inhibitory activity against methicillin resistant- and sensitive-strains of *S. aureus* (Fracchia et al., 2010; Lee et al., 2016). In addition to bacterial biofilm, fungal biofilm associated with *Candida* pathogens is responsible for serious *C. albicans* infections linked to biofilm formation on medical devices. One study showed that *Lactobacillus* biosurfactants displayed high anti-adhesive biofilm formation properties against *C. albicans* and also prevented biofilm formation of *L. monocytogenes*, *Salmonella arizonae*, *E. coli*, and *S. aureus* (Fracchia et al., 2010).

### Others

Natural products can also act as antiparasitic agents. The avermectins (14; Figure 3 and Table 1) and the derivative ivermectin have shown antiparasitic activity by significantly lowering the incidence of onchocerciasis and lymphatic filariasis (Shen, 2015). Spinosad and milbemycin also have insecticidal activity. Spinosad is a combination of spinosyn A and D, which are both produced by *Saccharopolyspora spinosa* and have insecticidal activity against livestock external parasites via the disruption of nicotinic acetylcholine receptors (Sanchez et al., 2012). Milbemycin is an isolated fermentation product of *S. hygroscopicus* subsp. *aureolacrimosus* that acts as an insecticide and acaricide with GABAergic activity on the post-synaptic membranes of the inhibitory motor neurons of mites and arthropods through hyperpolarization and impeding neuronal signal transduction mechanisms (Copping and Duke, 2007). Mollemycin A 20 (15; Figure 3 and Table 1) is a first-in-class glycol-hexadepsipeptide-polyketide from a *Streptomyces* sp. and has antibacterial properties against certain Gram-positive and Gram-negative bacteria, as well as extremely potent antimalarial activity against drug sensitive and MDR *Plasmodium falciparum* clones (Blunt et al., 2016). Microbial natural products also function as enzyme inhibitors. Lipstatin (16; Figure 3 and Table 1) is a pancreatic lipase inhibitor produced by *Streptomyces toxytricini* that is used to combat obesity and diabetes by interfering with the gastrointestinal absorption of fat (Weibel et al., 1987). Lipstatin contains a beta-lactone structure that is likely responsible for irreversibly binding to the active site of lipase (Sanchez et al., 2012).

### Biological Activity of Microbial Biologics

**FIGURE 4:**
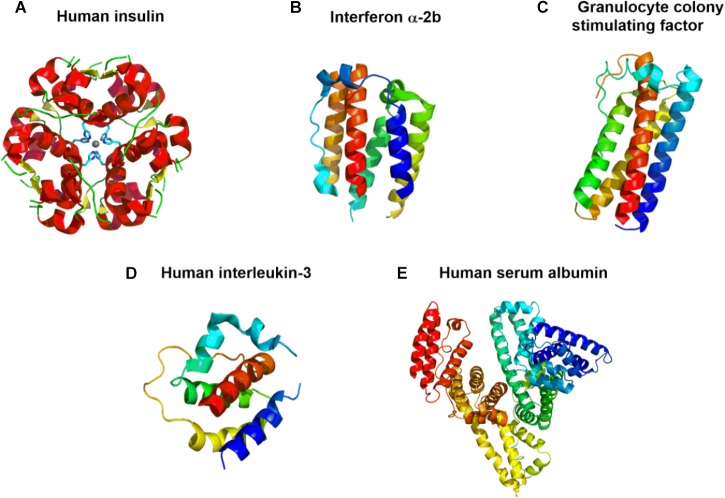
Crystal structures of **(A)** recombinant human insulin (Humulin^®^) (PDB 4F0N) (Favero-Retto et al., 2013); **(B)** interferon (IFN) α-2b (PDB 3SE3) (Thomas et al., 2011); **(C)** granulocyte colony growth factor pegfilgrastim (Neupeg^®^) (PDB 1HRG) (Hill et al., 1993); **(D)** human interleukin-3 (PDB 5UV8) (Broughton et al., 2018); and **(E)** human serum albumin (Recombumin^®^and Albucult^®^) (PDB 1AO6) (Sugio et al., 1999). The models are colored according to the sequence by a rainbow color from the N-terminus (blue) to the C-terminus (red).

Since Humulin^®^(Figure 4A) became the first recombinant biopharmaceutical as a treatment for diabetes (Johnson, 1983), additional FDA-approved microbial biologics have been produced by *E. coli*. Somatrem (Protropin^®^) and somatropin (Humatrope^®^) are used to treat children with growth hormone deficiency (Baeshen et al., 2015; Sanchez-Garcia et al., 2016). Another biopharmaceutical produced from *E. coli* is pegloticase (Krystexxa^®^) for the treatment of chronic gout and interferon (IFN) α-2b (Intron^®^A; Figure 4B) for the treatment of certain types of genital warts, malignant melanoma, hairy cell leukemia, follicular lymphoma, Kaposi’s sarcoma, and chronic Hepatitis B or C (Baeshen et al., 2015; Sanchez-Garcia et al., 2016). Top selling biopharmaceuticals of 2015 from microorganisms include insulin glargine (Lantus^®^) derived from *E. coli*, which functions as an insulin analog, and the pneumococcal vaccines (Prevnar^®^family) derived from *S. pneumoniae* and *Corynebacterium diphtheriae* (Jozala et al., 2016; Sanchez-Garcia et al., 2016). Biopharmaceuticals are also utilized for their antitumoral properties, such as the cytokines filgrastim (Neupogen^®^) and granulocyte colony stimulating factor pegfilgrastim (Neupeg^®^; Figure 4C), which are both derived from *E. coli*. Flgrastim stimulates hematopoiesis for bone marrow transplantation and cancer chemotherapy-induced neutropenia, whereas pegfilgrastim stimulates the differentiation, proliferation and activation of neutrophilic granulocytes for cancer chemotherapy-induced neutropenia (Sanchez-Garcia et al., 2016). Recombinant human interleukin-3 (hIL-3; Figure 4D) protein is a cytokine that regulates the differentiation and proliferation of the various cells of the immune system (Hercus et al., 2013). The hIL-3 protein is derived from *B. subtilis*, *B. licheniformis*, and *E. coli* and has utility as a laboratory reagent in hematology for cell cultures, differentiation studies and functional assays. It has shown that hIL-3 has potential in treating bone marrow failure, hematological malignancies, and can support engraftment after bone marrow transplantation (Westers et al., 2006). In addition, recombinant *Pfs48/45* is a disulfide-rich malaria transmission-blocking vaccine produced by *L. lactis* that provides immunization against malaria from *P. falciparum* (Song et al., 2017).

Recombivax is produced by *S. cerevisiae* and can prevent infection of all known subtypes of the Hepatitis B virus (Sanchez-Garcia et al., 2016). Some examples of currently approved protein therapeutics derived from yeast include human serum albumin (Recombumin^®^and Albucult^®^; Figure 4E), human insulin (Actrapid^®^) and primary immunization for infants born of Hepatitis B virus (HBV) surface antigen (Pediarix^®^), all of which are obtained exclusively from *S. cerevisiae* (McAleer et al., 1984; Ballance, 1999; Nielsen, 2013; Nandy and Srivastava, 2018). Recombinant human serum albumin is utilized to increase the shelf life of protein drugs by preventing physical and chemical degradation. Actrapid^®^is used to treat diabetes, and subcutaneous injections of Pediarix is designed for immunization against diphtheria, tetanus, pertussis, poliomyelitis, and infection caused by all known subtypes of HBV (Nandy and Srivastava, 2018). Ecallantide (Kalbitor^®^) is an FDA-approved recombinant peptide produced by *Pichia pastoris* for the treatment of hereditary angioedema (Sheffer et al., 2011). Additionally, anakinra (Kineret^®^) was approved in 2001 in the United States for rheumatoid arthritis (Baeshen et al., 2015). Anakinra is expressed in *E. coli* and functions as an IL-1 receptor antagonist that is effective and safe for patients with systemic-onset juvenile idiopathic arthritis, adult-onset Still’s disease, hereditary autoinflammatory syndromes, and Schnitzler’s syndrome (Kalliolias and Liossis, 2008; Jozala et al., 2016).

## Microbial Cell Factories

Selecting a suitable host strain is one of the most important aspects in the design of natural product and recombinant protein bioprocesses. We will review the characteristics of the microbial strains used to produce natural products and biologics in this section. We will also present the tools and strategies that facilitate engineering of the hosts as microbial cell factories for the production of biopharmaceutical compounds (Table 2).

**Table 2:** Comparison between different microbial host systems for production of recombinant proteins and natural products.

Microbial hosts	Advantages	Disadvantages	Compounds	References
**Gram-negative** *Escherichia coli*				
	• Fast growth • Simple culture procedures • Cost-effective • High versatility of the enterobacterium and its associated systems	• Lack of post-translational modifications (PTMs) • Risk of translational errors due to the presence of a large number of rare codons • Expensive and often challenging purification process	• Recombinant human insulin • Artemisinin • Erythromycin A • Somatrem • Somatropin • Pegloticase • Insulin glargine • Pneumococcal vaccines • Filgrastim • Pegfilgrastim • Human serum albumin • Hepatitis B virus immunization • IFN α-2b • IL-6	Johnson, 1983; Abdin et al., 2003; Chang et al., 2007; Zhang et al., 2010; Ferrer-Miralles and Villaverde, 2013; Baeshen et al., 2015; Jozala et al., 2016; Sanchez-Garcia et al., 2016
**Gram-positive** *Lactococcus lactis*				
	• Simplified downstream purification processes • Absence of endotoxins or unwanted glycosylation of proteins • Generally recognized as safe (GRAS) • Lack of secreted heterologous proteins degradation • Nisin-controlled gene expression system • Heterologous protein delivery in foodstuff or in the digestive tract	• Per liter secretion generally less robust than *Bacillus* sp. • AT-rich codon usage and/or the distribution of rare codons	• Nisin A • Pfs48/45 • Enterocin A • Pediocin PA-1 • IL-2 • IL-6 • Peanut allergen • Tetanus toxin fragment C • Transforming growth factor-β1	Steidler et al., 1998; Drouault et al., 2000; Martínez et al., 2000; Le Loir et al., 2005; Mierau and Kleerebezem, 2005; Glenting et al., 2007; Morello et al., 2008; Li and Vederas, 2009; Linares et al., 2010; Gyawali and Ibrahim, 2014; Bermúdez-Humarán et al., 2015; Li et al., 2015; Song et al., 2017
*Streptomyces* sp.	• Rapid growth • Abundant supply of secondary metabolite precursors • Ability to produce natural products. • Efficient protein secretion system such as Sec pathway and twin-arginine-translocation (Tat) pathway • Well-developed genetic manipulation	• Forms pellets or clumps • Low protein yield	• Streptomycin • Pikromycin • Kanamycin • Nystatin • Anthracyclines • Rapamycin • FK506 • Strepsesquitriol • Salinamides A and B • Cahuitamycin • Actinomycin D • Milbemycin • Mollemycin A • TNF α • hIL-10 • Streptokinase • IL-1β • IFN-α1 • Transforming growth factor-α • IL-2 • IFN-α2b • Tetracycline • Daptomycin • Chloramphenicol	Stanley and English, 1965; Kaslow et al., 1994; Trischman et al., 1994; Mann, 2001; Jung et al., 2006; Copping and Duke, 2007; Park et al., 2008, 2016; Vrancken and Anne, 2009; Fracchia et al., 2010; Anné et al., 2012; De Lima Procópio et al., 2012; Sanchez et al., 2012; Yang et al., 2013; Kim et al., 2015; Blunt et al., 2016; Jozala et al., 2016; Gao et al., 2017
*Bacillus* sp.	• Outstanding fermentation properties and protein production yield (20–25 g per liter) • Completely free toxin production • Flexibility for genetic engineering • Presence of proteome secretory pathway	• Primarily used in Enzyme production. • Plasmid instability • Presence of proteases: leads to difficulty in the production of recombinant proteins.	• Ieodoglucomide C • Ieodoglycolipid • Bacillomycin D and L • Alkaline cellulose • Alkaline protease • Alkaline α-amylase • hIL-3 • Fengycin • IL-1β • IFN-α2 • Staphylokinase • Iturins • Surfactin	Palva et al., 1983; Peypoux et al., 1984; Bellini et al., 1991; Kim et al., 2001; Westers et al., 2006; Deleu et al., 2008; Chang et al., 2011; Van Dijl and Hecker, 2013; Wang T. et al., 2015; El-Hossary et al., 2017
**Fungi/yeast** *Saccharomyces cerevisiae*				
	• Fast growth rate • Technically practical • Cost-effective • Ability to generate post-translational modification as *O*-linked glycosylation, phosphorylation, acetylation, and acylation • Advanced fermentation science	• *N*-linked glycosylation patterns differ from higher eukaryotes • Lack some required precursor pathways • Codon usage is biased toward A + T	• Human serum albumin • Recombinant human insulin • Hepatitis B virus immunization • Artemisinic acid • Paclitaxel • hIL-6 • Insulin aspart • Pfs25 • Sapogenin • Saponin	McAleer et al., 1984; Guisez et al., 1991; Kaslow et al., 1994; Ballance, 1999; Ferrer-Miralles et al., 2009; Nielsen, 2013; Paddon et al., 2013; Baeshen et al., 2014; Ding et al., 2014; Meehl and Stadheim, 2014; Moses et al., 2014; Kung et al., 2018; Nandy and Srivastava, 2018
*Aspergillus* sp.	• GRAS status • Tolerate extreme cultivation conditions • Degrade and utilize diverse biopolymers, allowing cultivation on renewable resources • Major Source of citric acid production	• Production of mycotoxins (alpha toxins) • Many host proteases • Freely dispersed filaments or highly compact pellets formed during submerged fermentations	• Immunoglobulin G1(κ) • Antibodies and Fab′ fragment • Bicoumanigrin • Aspernigrin B • Lactoferrin • Enniatin • Human IL-2 • Human IL-6 • Phytase • L-asparaginase • Lovastatin • Tryptostatin B	Gaffar and Shethna, 1977; Carrez et al., 1990; Hiort et al., 2004; Papagianni, 2004; Ward et al., 2004; Grimm et al., 2005; Maheshwari, 2006; Pel et al., 2007; Maiya et al., 2009; Meyer et al., 2011; Cragg and Newman, 2013
*Hansenula polymorpha*	• GRAS status • Combined genetic manipulations, low cost screening. • Efficient fermentation properties, and protein modification • Ability to use and grow on methanol, glucose, or glycerol as its primary carbon sources • Thermo-tolerant	• The use of methanol creates hazardous conditions in lab use • Hyperglycosylation of heterologous products • Can lead to production instabilities due to sequence repetition on vector.	• IFNα-2a • Phytase • IL-6 • Human serum albumin • Human hemoglobin • HBV L-protein • Hepatitis B surface antigen	Janowicz et al., 1991; Gellissen et al., 1992; Hollenberg and Gellissen, 1997; Cox et al., 2000; Heijtink et al., 2002; Müller et al., 2002; Böer et al., 2007; Kunze et al., 2009; Celik and Calik, 2012


### Gram-Negative Bacteria

#### Escherichia coli

*Escherichia coli* has been seen as one of the optimal systems for the production of natural products because it is easily manipulated, highly productive, there is an availability of genetic tools to use with it and there is a deep knowledge of its physiology. Artemisinin, a sesquiterpene lactone endoperoxide from *Artemisia annua* L. plants, has strong antimalarial activity against the multi-drug resistant parasite *P. falciparum* (Abdin et al., 2003). Yet the synthesis of artemisinin is costly and low yields are isolated from the natural plant source. Researchers reported the production of approximately 24 mg/L of amorpha-4,11-diene (amorphadiene), an artemisinin precursor, by the expression of a codon-optimized synthetic amorphadiene synthase gene and the mevalonate pathway from *S. cerevisiae* in *E. coli*. Additionally, after further processing modifications and optimal conditions, they were able to produce 105 mg/L of artemisinic acid (Chang et al., 2007). However, there are some obstacles and limitations with *E. coli* as a dominant host in natural product biosynthesis. *E. coli* requires extensive genetic manipulation and lacks native natural product biosynthetic machinery and/or precursors. An example is phosphopantetheinyl transferase, which is responsible for the activation of the carrier protein domains of the PKSs and NRPSs. This enzyme must be introduced into *E. coli* to support of natural product biosynthesis (Zhang M.M. et al., 2016). There have been efforts to overcome these hurdles, such as the production of erythromycin A and its derivatives in the engineered *E. coli* strain (Zhang et al., 2010). The study generated two analogs through directed manipulation of polyketide biosynthesis in which variations were made to the deoxyerythronolide B synthase (DEBS) 1 and DEBS3 enzymes in order to utilize the multi-catalytic capability of the modular polyketide synthase (Zhang et al., 2010).

*Escherichia coli* has also been the pioneering host for recombinant protein production. To date, *E. coli* continues to be the first-choice microorganism for manufacturing recombinant proteins at laboratory and industrial scales. Its success is mostly due to its fast growth, simple culture procedures, cost-effectiveness, unusually high versatility, and the associated systems that make it adaptable to varying production demands (Ferrer-Miralles and Villaverde, 2013; Sanchez-Garcia et al., 2016). From 2004 to 2013, 24% of the biopharmaceuticals approved by the FDA and the European Medicines Agency were derived from *E. coli* (Baeshen et al., 2014). Currently, biopharmaceuticals produced from *E. coli* are used in the treatment of diabetes, growth hormone-deficiency in children, leukemia, gout, and many other therapeutic indications as previously discussed in Section “Biological Activity of Microbial Biologics” (Baeshen et al., 2015). A major concern when using *E. coli* as a production platform is the lack of post-translational modifications (PTMs) present in most eukaryotic proteins; lacking PTMs can lead to protein products being insoluble, unstable, or inactive (Ferrer-Miralles et al., 2009). However, it is possible to add synthetic PTMs to generate versions of the protein that are more stable than the original naked product (Ferrer-Miralles et al., 2009). Examples of this include pegylated products, like human growth hormone, stimulating factor, IFNs α-2a and α-2b, (Ferrer-Miralles et al., 2009). Additionally, there is a risk of translational errors due to the presence of a large number of rare codons that appear in human genes that are different from those occurring in *E. coli* genes. Even at low levels, these errors may cause an impact on the tertiary structure, premature termination of protein synthesis or amino acid misincorporation which results in low protein expression (Gustafsson et al., 2004). One strategy to bypass the issue with codon bias is to synthesize the whole human gene based on codon usage in *E. coli* through site-directed mutagenesis, which is currently a preferred method; however, it is limited by the high cost of production and time consumption (Sørensen and Mortensen, 2005). An alternative method that is less time consuming utilizes the co-transformation of *E. coli* strains with a plasmid(s) containing a gene encoding the tRNA cognate to the rare codons (Dieci et al., 2000). Increasing the copy number allows for *E. coli* to be manipulated to match the codon usage frequency in heterologous genes (Dieci et al., 2000). Currently, there are numerous commercial *E. coli* strains available that harbor plasmids containing gene sequences encoding the tRNA for rare codons, such as BL21(DE3) CodonPlus-RIL, BL21(DE3) CodonPlus-RP and Rosetta (DE3) (Baeshen et al., 2015). Another common problem associated with recombinant protein expression in *E. coli* involve inclusion body formation, which refers to insoluble and inactive protein aggregates (Hartley and Kane, 1988). *E. coli* producing recombinant proteins have the ability to assemble in cells and form conglomerates of inclusion bodies as well as result in erroneous protein folding which hinder the extraction of proteins directly from the cell leading to costly purification of proteins (Zweers et al., 2008). Inclusion bodies formed from lack of proportion between protein solubilization and aggregation can be resolved by combining the desired protein with a solubility enhancer fusion partner acting as an intrinsic chaperone in order to ensure the production of soluble recombinant proteins (Rosano and Ceccarelli, 2014). The fusion of maltose-binding protein to polypeptides such as human tissue inhibitor of metalloproteinase and p16 improved their solubility significantly in *E. coli* (Kapust and Waugh, 1999).

### Gram-Positive Bacteria

#### Lactococcus lactis

*Lactococcus lactis* is becoming an attractive alternative in genetic engineering for the production of various recombinant proteins. Unlike *E. coli*, which uses intracellular production strategies that involve expensive and often challenging purification processes, *L. lactis* utilizes extracellular secretion system. This is because *L. lactis* has a monolayer cell wall that allows direct secretion into the extracellular environment (Schneewind and Missiakas, 2012). The presence of exported proteases such as HtrA in *L. lactis* contributes to recombinant protein production by minimizing the destruction of heterologous proteins in the medium (Morello et al., 2008; Song et al., 2017). Additionally, *L. lactis* does not generate undesired glycosylation of protein, is generally recognized as safe (GRAS), does not produce endotoxins, and has probiotic properties (Singh et al., 2018). Another advantage of *L. lactis* includes a lack of inclusion body formation (Theisen et al., 2017). There is a diverse selection of cloning and inducible expression vectors available for use with this host that are compatible with large-scale upstream and downstream processes (Song et al., 2017).

*Lactococcus lactis* has been used for centuries in the fermentation of food, especially in cheese, yogurt, and sauerkraut because of its production of nisin (Song et al., 2017; Singh et al., 2018). Beyond the food industry, lactic acid is used as an emulsifier and moisturizing agent in the cosmetic industry and as an important raw material in the pharmaceutical industry (Papagianni, 2012). The *L. lactis* host has also been chosen after researchers had unsuccessfully attempted to obtain correct conformation using a variety of other prokaryotic and eukaryotic recombinant protein expression systems. *L. lactis* has multiple advantages over *E. coli* for expression of 5′-methylthioadenosine/*S*-adenosylhomocysteine nucleosidase (*Pfs*) recombinant proteins, including the following: (1) codon-optimization of the recombinant gene is not necessary to achieve successful expression in *L. lactis*; (2) the recombinant protein is secreted into the *L. lactis* culture supernatant, which results in easier and less expensive down-stream processing, and (3) there is no lipopolysaccharide contamination in *L. lactis* expression (Singh et al., 2018). *L. lactis* has been used in the successful production of recombinant *Pfs48/45*, a vaccine candidate against *P. falciparum* (Song et al., 2017). GMZ2, a recombinant fusion protein expressed in *L. lactis*, is also a malaria vaccine candidate that has been shown to elicit high levels of active IgG antibodies with inhibitory activity against a broad range of *P. falciparum* strains (Jepsen et al., 2013). A recent study concluded phase 2 trial of GMZ2 adjuvanted with aluminum hydroxide in a cohort of 1,849 children revealed GMZ2 as well tolerated with modest efficacy (Sirima et al., 2016). Not only is *L. lactis* being utilized for the production of recombinant proteins for vaccines, but the host is also being tested as a factory for antigen production, allowing the bacteria to function as live vaccines. Using *L. lactis* as a vaccine carrier is beneficial because it can induce both mucosal and systemic immune responses, has adjuvant properties and is free from the risks associated with the use of conventional attenuated live pathogens, such as *Salmonella* species and *Mycobacterium* species (Song et al., 2017). However, while *L. lactis* has been studied against an array of antigens from various pathogens, there is no current live vaccines under clinical trial which may be due to a lack of containment strategies (Bahey-El-Din, 2012). Without a plan for containment, studies on live *L. lactis* vectors risk the chance of proliferation and dispersion. An additional limitation of AT rich *L. lactis* as a cell factory is due to codon usage as well as distribution of rare codons to express heterologous genes (Mierau and Kleerebezem, 2005).

#### *Streptomyces* Species

Another major species that has shown promise as a cell factory through its wide production of natural products and biologics is *Streptomyces*. This Gram-positive bacterium has been studied for more than 70 years and has proven to be of great use in biotechnology due to its ability to produce natural products acting as antibiotics, anticancer agents, and immunosuppressants (Yoo et al., 2015). Some examples include tetracycline, daptomycin, and chloramphenicol (De Lima Procópio et al., 2012). There are many species of *Streptomyces* currently to produce various natural products and biologics. Among the *Streptomyces* species, *Streptomyces coelicolor*, *Streptomyces lividans*, *Streptomyces albus*, and *S. venezuelae* are favored heterologous hosts to produce specialized metabolites due to the relative ease of their genetic manipulation, the availability of their genome sequences, and the abundant supply of their natural substrates (Park et al., 2010). *Streptomyces* has also been used to produce a wide array of heterologous proteins of both eukaryotic and prokaryotic origin (Gilbert et al., 1995) because *Streptomyces* has well-developed genetic manipulation and fermentation technologies as well as efficient protein secretion systems such as the secretory (Sec) pathway and the twin-arginine-translocation (Tat) pathway (Hamed et al., 2018). The Sec-pathway catalyzes the translocation of unfolded proteins while the Tat pathway allows for the export of folded proteins across the cytoplasmic membrane (Natale et al., 2008). Tumor necrosis factor (TNF) α and human interleukin (hIL) 10 are able to be expressed in both the Sec- and Tat-pathways (Schaerlaekens et al., 2004). In particular, *S. lividans* could be the ideal *Streptomyces* host due to limited restriction-modification systems and low endogenous protease activity (Nakashima et al., 2005). Streptokinase (Pimienta et al., 2007), transforming growth factor-α (Taguchi et al., 1995), IL-2 (Bender et al., 1990) and many other proteins have been successfully expressed and secreted from *S. lividans.* However, aside from its efficient secretory pathways, when in culture, *Streptomyces* grows as mycelial networks, leading to the formation of pellets or clumps (Van Dissel et al., 2015). These pellets are unappealing from an industrial standpoint because of mass-transfer problems, slow growth, and culture heterogeneity which leads to lower product yield (Van Dissel et al., 2015).

#### *Bacillus* Species

*Bacillus* species are some of the most popular species used in enzyme production. It accounts for roughly 50% of enzymes market within the industrial sphere (Barros et al., 2013). Certain species, like *B. subtilis*, *Bacillus amyloliquefaciens*, and *B. licheniformis* are favored because of their outstanding fermentation properties, high protein production yield, and their completely toxin-free production (Van Dissel et al., 2015). The fermentation capacity of *Bacillus* species in acid, neutral, and alkaline pH ranges in addition to thermophiles accounts for the prolific production of enzymes that have desirable temperature, pH, and stability, which makes them appealing for specific use in various industries (Schallmey et al., 2004). *Bacillus* species are known for their production of iturins and fengycin which have antifungal activity as well as surfactin for its function as a surfactant (Wang T. et al., 2015).

Among these species, *B. subtilis* is the most widely studied due to (1) its flexibility during genetic engineering, (2) its naturally high secretory capacity, (3) its ability to produce valuable antibiotics, such as bacillomycins D-L and bacitracin, and (4) its ability to produce enzymes, such as stable alkaline cellulase, alkaline protease and alkaline α-amylase. It may also elicit better folding conditions, leading to the prevention of inclusion bodies (Peypoux et al., 1984; Van Dijl and Hecker, 2013). In addition, its ability to adapt to varying environmental conditions as well as its classification as toxic free GRAS has contributed tremendously to its success in the industrial platform (Baysal and Yıldız, 2017). *B. subtilis* as an endotoxin free host amplified its utilization in the production of sterile recombinant and therapeutic proteins expression as compared to *E. coli* which could have potential contamination due to the lipopolysaccharide endotoxins (Wang et al., 2013). For instance, *B. subtilis* and *Bacillus megaterium* were the preferred hosts over *E. coli* in the production of bioengineered heparin in order to diminish toxin contamination (Wang et al., 2013). Moreover, *B. subtilis* is able to produce high yield in enzyme as it secretes the enzymes straight into the fermentation medium due to the absence of outer membrane which allows easy recovery of purified proteins from the medium into their active form (Zweers et al., 2008). It has the capacity to secrete about 20–25 g/L of enzymes into the medium (Schallmey et al., 2004). Enzymes produced by *B. subtilis* has a wide variety of applications ranging from pharmaceutical, leather, detergent, food, and waste management industries (Singh et al., 2016).

Aside from enzyme production, cytokines like hIL-3 have been produced by *B. subtilis* and *B. licheniformis*. The production of hIL-3 has been tested in various host organisms, including *E. coli*. However, the production of IL-3 within other organisms has often exhibited problems, such as insolubility or the degradation of produced hIL-3. This led to the use of *B. licheniformis* and *B. subtilis* to minimize such complications. The production of hIL-3 in *B. licheniformis* was engineered to lack four C-terminal residues, resulting in a fully active hIL-3 protein. However, residual proteolytic degradation of some hIL-3 still occurred, leading to use *B. subtilis* to achieve complete folding and full biological activity of hIL-3 (Westers et al., 2006).

Among the *Bacillus* species, *Bacillus thuringiensis* is best known for being widely used within the agricultural industry due to its insecticidal properties through its production of parasporal crystals during the stationary phase of its growth cycle (Höfte and Whiteley, 1989; Schnepf et al., 1998). Upon ingestion, the parasporal crystals are solubilized in the midgut of insects, resulting in the release of protoxin proteins known as δ-endotoxins, leading to the formation of pores throughout the cell membrane (Höfte and Whiteley, 1989; Gill et al., 1992). Parasporal proteins also have selective cytotoxicity against liver and colon cancer cells while leaving normal cells unharmed (Ito et al., 2004).

However, the use of *Bacillus* has been restricted to mainly enzyme production and non-recombinant protein therapeutics, which may be due to the lack of associated expression vectors, plasmid instability and the presence of native proteases (Westers et al., 2004). Despite *B. subtilis* success as the industrial workhorse, it has its drawbacks in the production of heterologous proteins. Heterologous protein yield could diminish when using the *Bacillus* as a host due to the proteolytic destruction of foreign protein by host secreted extracellular proteases (Nijland and Kuipers, 2008). Efforts have been made to improve the production of heterologous protein by manipulating the expression of proteins involved in the post translocation phase resulting in amplified levels of heterologous protein secretion (Vitikainen et al., 2005). In contrast to *E. coli*, the absence of distinguished and controllable promoters in *B. subtilis* interferes with successful expression of heterologous genes resulting in inefficient production of heterologous proteins (Schallmey et al., 2004).

### Yeast/Fungi

#### Saccharomyces cerevisiae

As with *E. coli*, *S. cerevisiae* has been extensively used for the production of recombinant human insulin since the early 1980s, and it currently produces half of the world’s supply of insulin (Meehl and Stadheim, 2014). *S. cerevisiae* is preferred because it is also cost-effective, fast growing, technically practical, and is amenable to large-scale fermentation in bioreactors. Yeast is often utilized as a cell factory when the target protein is not produced in a soluble form in prokaryotic systems or when a specific PTM cannot be produced or added to the naked product. *S. cerevisiae*, as with other yeast species, can perform many PTMs such as *O*-linked glycosylation, phosphorylation, acetylation, and acylation, which allows recombinant proteins to be expressed in a soluble, correctly folded, and functionally active form (Ferrer-Miralles et al., 2009; Baeshen et al., 2014). Some examples of currently approved protein therapeutics derived from yeast include human serum albumin, insulin, and primary immunization for infants born of HBV surface antigen, all which are obtained in *S. cerevisiae* (McAleer et al., 1984; Ballance, 1999; Nielsen, 2013; Nandy and Srivastava, 2018). However, the significant drawback to producing protein therapeutics from *S. cerevisiae* is that higher eukaryotes have a different pattern of *N*-linked glycosylation, which can decrease the half-life and cause hyper-immunogenicity, leading to less effective therapeutics (Ferrer-Miralles et al., 2009). In recent years, there have been some advances in limiting *S. cerevisiae* hypermannosylation. These yeast glycoengineering techniques involve two main stages, (1) the removal of yeast hypermannosylation and (2) the conversion to complex glycoforms containing terminal sugars, such as *N*-acetylglucosamine, galactose, or sialic acid. These recent reports on yeast *N*-glycan humanization indicate a move from the proof of concept phase to implementation (Meehl and Stadheim, 2014).

Another current area of study is the production of plant and microbe-derived secondary metabolites. Due to the structural complexity of secondary metabolites, chemical synthesis is an inefficient route for large-scale production, and fermentation is the main mode for economic commercial production of pharmaceutically useful natural products (McDaniel et al., 2001). *S. cerevisiae* could be an ideal candidate as a microbial host as it boasts relatively rapid growth, and it is accompanied by highly developed genetic tools and advanced fermentation science. Like *E. coli*, *S. cerevisiae* has been shown to be an outstanding production host for artemisinic acid, a precursor to the antimalarial agent artemisinin, with a high productivity (25 g/L) that led to the industrial production of semi-synthetic artemisinin beginning in 2013 (Paddon et al., 2013; Kung et al., 2018). Research has also produced the paclitaxel (Taxol^®^) precursor taxadiene (∼73 mg/L) by engineering the taxol biosynthetic genes in *S. cerevisiae* (Ding et al., 2014). Besides plant secondary metabolites, *S. cerevisiae* has generated a remarkable titer (1.7 g/L) of microbial polyketide 6-methylsalicylic acid in un-optimized shake-flask fermentations. In addition, *S. cerevisiae* has been developed as a heterologous host to express fungal cryptic BGCs and their respective metabolites. In this study, 30 *ADH2*-like promoters in *Saccharomyces* species were developed as tools for expression of 41 heterologous BGCs, 22 of which were capable of producing heterologous compounds, including novel compounds. For example, BGCs derived from basidiomycete were found to produce *N*-, *S*-bis-acylated amino acids and a leucine *O*-methyl ester with an additional polyketide chain amidated to the amino ester (Harvey et al., 2018). However, barriers still exist to the heterologous production of complex molecules. This includes the production of polyketides in *S. cerevisiae*, as the host lacks some required polyketide precursor pathways, its codon usage is biased toward A + T (most microbial polyketide producers have high G + C genome content) and it lacks the appropriate endogenous phosphopantetheinyl transferase capable of the necessary PTMs (Mutka et al., 2006).

#### *Aspergillus* Species

Multicellular filamentous fungi, such as *A. niger* and *Aspergillus oryzae*, can also offer great potential in the production of a desired substance by fermentation due to the following reasons: (1) they are well-characterized GRAS organisms, (2) are amenable to scaled-up fermentation, (3) can be genetically engineered, (4) they are capable of secreting a high level of proteins and (5) can withstand adjustable cultivation conditions, including temperature (5–45°), pH (2–11), salinity (as much as 34%), water activity (0.92–0.98), and both nutrient rich and poor environments (Maheshwari, 2006; Meyer et al., 2011).

*Aspergillus niger* has been predominantly used for industrial-level production of citric acid through anaerobic fermentation process. As a weak acid, citric acid can serve as a natural preservative, flavoring agent in food and beverages, antioxidant, acidulant, pH-regulator, chelating agent or vegetable rinse, as well as comparable applications in the pharmaceutical and cosmetics industries (Cairns et al., 2018). Due to its wide variety applications, its ease of production, and economical potential of citric acid, the market of citric acid has become one of the fastest-growing regions of the food additives market due to the rising demand: according to estimations, in 2007, the market value of citric acid exceeded $2 billion in 2014 and is predicted to rise to $3.6 billion by 2020 (Show et al., 2015; Cairns et al., 2018). Phytase is another example that have produced by *A. niger* fermentation (Papagianni et al., 1999). The significance of phytase enzymes lie in its ability to interact with the nutrient rich compounds known as phytate. Phytate, or phytic acid, is a common energy source found in oilseeds, cereals, and legumes, which are used in providing nutrition to animal feeds (Schlemmer et al., 2009). Combining citric acid with phytase has also been shown to enhance phytase activity on phytate, producing greater nutrient outcomes in tested animals (Boling et al., 2000). In addition, two different humanized immunoglobulin G1(κ) antibodies and an Fab′ fragment were produced by *A. niger*, and the antibodies were successfully secreted into the culture supernatant (Ward et al., 2004). *Aspergillus* strains have been also used to produce the human iron-binding glycoprotein lactoferrin and hIL-2 with the yields of 25 and 150 mg/L, respectively (Maheshwari, 2006). Bicoumanigrin with cytotoxic activity against human cancer cell lines and aspernigrin B with neuroprotective effects have both been isolated from *A. niger* (Hiort et al., 2004). The product spectrum of *Aspergillus* species is not restricted to biologic molecules. As an example, a novel cyclic peptide compound, KK-1, with potent antifungal activity was produced in *A. oryzae* by introducing gene clusters spanning approximately 40 kb from the plant-pathogenic fungus *Curvularia clavata* into the genome of *A. oryzae*. Although the amount of KK-1 produced by the host was lower than that produced by the original producer *C. clavata*, this result indicated that a gene twice as large as the largest native gene in *A. oryzae* could be successfully expressed (Yoshimi et al., 2018). Furthermore, when the industrial fungus *A. niger* was engineered as a heterologous host, it produced high titers (up to 4,500 mg/L) of enniatin belonging to non-ribosomal peptides with antibacterial, antiviral, and anticancer activities (Richter et al., 2014).

#### Hansenula polymorpha

Another industrially important yeast species that has shown promise in the production of peptides is *Hansenula polymorpha* (Gellissen et al., 1992; Boer et al., 2007). *H. polymorpha* is a methylotrophic yeast species with the ability to use and grow on methanol, glucose, or glycerol as its primary carbon source (Gellissen et al., 1992). Like *S. cerevisiae* and *Aspergillus* species, *H. polymorpha*, classified as GRAS organism, does not harbor pyrogens, toxins, pathogens, or viral inclusions (Ubiyvovk et al., 2011). It is distinguished by very high cell densities in bioreactors and characterized by simple cultivation mode in inexpensive growth media. For example, *H. polymorpha* has allowed for cost-effective production of phytase through cheap carbon sources (Mayer et al., 1999). It possesses well-established genetic tools such as strong regulatory and constitutive promoters, which consequently give high product yield (Van Dijk et al., 2000). It also has thermotolerance properties, making *H. polymorpha* successful in crystallographic studies and in the production of recombinant proteins like IFN-2α, IL-6, recombinant human serum albumin, glucose oxidase, and catalase (Kunze et al., 2009; Celik and Calik, 2012). A notable feature of *H. polymorpha* is the significant growth of peroxisomes when grown on methanol which allows for high storage capacity of soluble proteins. The lack of protein modifying enzymes in the matrix of peroxisomes also provides an advantage for the development of heterologous proteins that are susceptible to proteolytic degradation (Van Dijk et al., 2000). Furthermore, the host has been used to produce L antigens found on the HBV viral envelope in attempt to produce the HBV vaccine. The L protein produced by *H. polymorpha* has increased stability in comparison to other yeast species, such as *S. cerevisiae* and *P. pastoris* (Janowicz et al., 1991). In addition to its use in vaccine production, *H. polymorpha* is also used in the production of human hemoglobin through the use of a single expression vector (Hollenberg and Gellissen, 1997). However, hyperglycosylation has been observed as a main drawback of *H. polymorpha* to produce heterologous products (Müller et al., 1998).

## Efforts in Product Improvements and Generation of New Analogs

**FIGURE 5:**
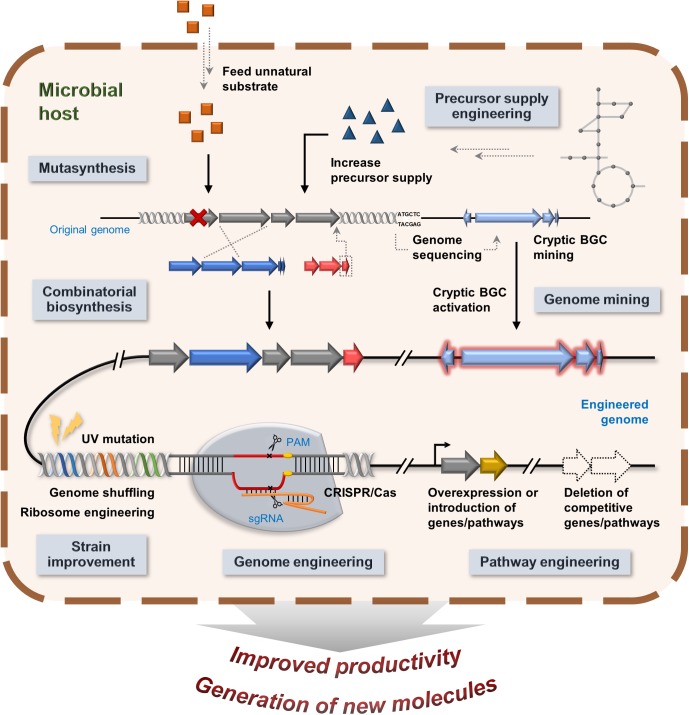
An overview of multiple strategies of product improvements and generation of new analogs.

There are multiple approaches that have been taken to advance product improvement for microbial natural products and biologics. This section will discuss efforts to combat the challenges of production of natural products and its analogs, including strain improvement, increasing precursor supply, pathway engineering, combinatorial biosynthesis, and genome mining (Figure 5).

### Strain Improvement

Whole-genome shuffling is a process that utilizes the advantages of the multi-parental crossing allowed by DNA shuffling with the genome recombination normally associated with conventional breeding (Zhang et al., 2002). Genome shuffling has been successfully improved the titers of variety of microorganisms. For example, two strains of *Streptomyces fradiae* generated from two rounds of genome shuffling were able to produce up to a ninefold increase in antibacterial tylosin production in comparison to the initial strain (Zhang et al., 2002). Using genome shuffling in a combination of protoplast fusion, mutant strain of *S. cellulosum* GSUV_3-205_ generated a 130-fold increase (104 mg/L) in production of epothilone when compared to starting strain *S. cellulosum* So0157-2 (0.8 mg/L) (Gong et al., 2007). Ribosome engineering is also a method useful in increasing secondary metabolite production titer and productivity (Sun and Alper, 2015). Studies demonstrate that *rpoB* mutations are effective in activating silent and poorly expressed secondary metabolite biosynthetic gene clusters (BGCs) at the transcriptional level in *S. griseus*, *S. coelicolor*, and *S. erythraea* (Ochi and Hosaka, 2013). For example, the H437R mutant of *rpoB* from *S. erythraea* was screened for drug resistance and was found to have an increased production of erythromycin (Tanaka et al., 2013; Ochi et al., 2014). Another study found a 37-fold increased production of avilamycin in a recombinant *Streptomyces viridochromogenes* strain due to a mutation in ribosome protein S12 (*rps12*) acquired through a combination of gene shuffling and ribosome engineering (Lv et al., 2013).

### Engineering Precursor Supply

Precursor supply is defined as the enhancement of the availability of primary metabolites or molecules derived from primary metabolism involved in the biosynthesis of natural products (Shiba et al., 2007). Precursor supply engineering can be achieved by manipulating either the pathways or enzymes involved with the precursor supply. Malonyl-CoA and methylmalonyl-CoA are the most commonly used and metabolically available precursors for the biosynthesis of polyketides. One study found that supplying methyl oleate enhanced the internal concentration of methylmalonyl-CoA, which is a biosynthetic precursor for FK506, and led to a 2.5-fold increase in FK506 production in *Streptomyces clavuligerus* CKD1119 (Mo et al., 2009). In another study, propionyl-CoA carboxylase with supplementation of propionate was found to effectively increase methylmalonyl-CoA and rapamycin titers in the mutant strain *S. rapamycinicus* UV_2-2_ induced by ultraviolet mutagenesis in comparison to wild-type strain (7 ∼ 8 mg/L) (Jung et al., 2011). The mutant strain was found to have a 3.2-fold improvement (23.6 mg/L) in comparison to wild-type strain *S. rapamycinicus* ATCC 29253 (7 ∼ 8 mg/L) (Jung et al., 2011). Further, Méndez and coworkers improved precursor metabolite pools for the production of the antitumor polyketide mithramycin in *Streptomyces argillaceus* by increasing the precursor supply of malonyl-CoA and glucose-1-phosphate (Zabala et al., 2013). Several classes of natural products utilize aromatic amino acids or other metabolites derived from the shikimate pathway as precursors, including flavonoids, alkaloids, polyketides, and non-ribosomal peptides (Knaggs, 2001). The production of the vancomycin analog balhimycin was increased 2.5-fold in *Amycolatopsis* sp. Y-89,21022. This was achieved by increasing the non-ribosomal peptide precursor 3-deoxy-D-arabino-heptulosonate7-phosphate synthase, the first enzyme in the shikimate pathway (Thykaer et al., 2010). In addition, manipulating key enzymes that direct carbon flux through core biochemical pathways involved in glucose, fatty acid, and amino acid metabolism can increase biosynthetic precursor pools. A study on the modulation of carbon flux between the pentose phosphate pathway and the glycolysis pathway found that a deletion of phosphofructokinase isoenzymes led to the enhanced production of antibiotics actinorhodin and undecylprodigiosin in *S. coelicolor* by increasing carbon flux through the pentose phosphate pathway (Borodina et al., 2008).

Precursor supply engineering has been successfully used to produce most of the major classes of natural products, with application to heterologous producing strains as well as native producers. When the native host is slow-growing or cannot be easily manipulated genetically, this process could be performed effectively in an appropriate heterologous host. A study bypassed the native deoxyxylulose 5-phosphate pathway and instead introduced the mevalonate pathway from *S. cerevisiae* to *E. coli*, which allowed for an increased production of amorpha-4,11-diene which is a precursor to antimalarial artemisinin (Martin et al., 2003). Combined approach of exogenous supplementation and engineering of intracellular pathway responsible for precursors can be also performed in a heterologous host. The biosynthetic process of the hybrid non-ribosomal peptide-polyketide yersiniabactin was known to rely on the supply of salicylate, L-cysteine, *S*-adenosyl-L-methionine, malonyl-CoA, and NADPH. When exogenous cysteine was fed to the culture of *E. coli* harboring yersiniabactin BGC and an additional set of genes (*hmwp1-2*) responsible for yersiniabactin precursor biosynthesis was introduced, the yersiniabactin production in *E. coli* was boosted to approximately 175 mg/L (Ahmadi and Pfeifer, 2016).

Precursor engineering strategy can be also employed to increase recombinant protein production by reducing unwanted by-products. One of the primary obstacles observed in high cell density cultivations of *E. coli* for the production of recombinant proteins is the formation of acetate, which is a by-product caused by an excess influx of carbon during aerobic fermentation. This acetate accumulation hampers cell growth and recombinant protein formation, even at low concentrations (Waegeman and Soetaert, 2011). A number of engineering approaches have focused on minimizing acetate formation in order to enhance recombinant protein production in *E. coli*. When a heterologous anaplerotic pyruvate carboxylase from *Rhizobium etli* is overexpressed in *E. coli*, the resulting strain had a 57% reduction in acetate formation and a 68% increase in β-galactosidase production (March et al., 2002). It is also possible to combine different strategies to reduce the formation of undesired by-products, including acetate. For example, one study found that a mutant *E. coli* strain containing a defective acetate pathway and an overexpressed phosphoenolpyruvate carboxylase-encoding *ppC* reduced acetate and other by-product formation and produced five time more β-galactosidase activity when compared the wild type strain (De Mey et al., 2010).

### Pathway Engineering

Metabolic pathway engineering can be performed in the native host through repetitive gene expression, gene deletion, and introduction of new genes to enhance production of natural products (Pickens et al., 2011). For example, overexpression of the 4–12 tandem copies of the actinorhodin cluster resulted in a 20-fold increase in actinorhodin production in *S. coelicolor* (Murakami et al., 2011). Additionally, a *S. hygroscopicus* strain with 3–5 tandem copies of the 40 kb validomycin A cluster showed a 34% increase in production and a maximum titer of approximately 20 g/L (Zhou et al., 2014). Deletion of genes may be useful to eliminate competing pathways that may siphon off important precursors or intermediates, or simply contribute to an unnecessary use of cellular resources which result to improve yields of products of interest. During *in vivo* bioconversion of lovastatin intermediate monacolin J to simvastatin using *E. coli* expressing heterologous acyltransferase LovD, it was found that *E. coli* could unexpectedly hydrolyze the synthetic thioester substrate. The responsible hydrolase BioH was knocked out to improve simvastatin production (Xie et al., 2007). The regulatory component of the pathway can be manipulated to enhance production of the resulting natural product. Negative regulation by pathway specific repressors can help regulate secondary metabolite pathways. For example, one study improved the titer by 100-fold of antibiotics platensimycin (323 mg/L) and platencin (255 mg/L) through the inactivation of a gene encoding protein PtmR1 belonging to GntR family of transcriptional repressors (Smanski et al., 2009). On the other side of the spectrum, *Streptomyces* antibiotic regulatory protein (SARP) is a positive regulator of antibiotic production (Chen et al., 2010). Overexpression of SARPs and/or increasing SARP gene dosage using multi-copy plasmids has been demonstrated to increase production titers. Overexpression of *mgsA* or *chxA*, SARP family members that are positive regulators for the iso-migrastatin and cycloheximide biosynthetic machinery, respectively, in *Streptomyces amphibiosporus* ATCC 53964 led to a fivefold increased production of antibiotic lactimidomycin (Zhang et al., 2016). Members of the large ATP-binding regulators of the LuxR (LAL) family also generally function as transcriptional activators, and constitutive overexpression of these LAL-type activators was found to increase production of rapamycin in *S. rapamycinicus* and FK506 in *S. tsukubaensis* (Kušèer et al., 2007; Mo et al., 2012).

Competing pathways can also be deleted to ensure the production of important precursors or intermediates and to save useful cellular resources. When deleting pathways, the idea is to create a host with a minimized genome to ensure the efficient production of necessary secondary metabolites. Deleting non-essential genes and directing cellular resources toward pathways that are essential for the survival and product biosynthesis can improve cellular efficiency and streamline biochemical production. For example, the genome of *Streptomyces avermitilis* was effectively minimized to 83% of its original size. When heterologous streptomycin gene cluster was introduced into the genome-minimized *S. avermitilis*, the resulting strain produced a higher titer of streptomycin than both the parent *S. avermitilis* carrying the same heterologous gene cluster and the native streptomycin producer *S. griseus* (Komatsu et al., 2010). However, large scale deletions may result in unintended effects as the complete workings of the cell are not yet entirely understood.

Similar approaches have been employed to improve the secretion capability and productivity of biologics. Engineering the protein trafficking pathway represents one successful approach to improve the secretion of heterologous recombinant proteins. For example, the secretion of the heterologous proteins human insulin precursor and α-amylase from *A. oryzae* in *S. cerevisiae* was improved by the over-expression of Sec1/Munc18 proteins, which are involved in the protein secretory pathway (Hou et al., 2012). Increasing the copy number of genes that are associated with protein secretion can also enhance protein secretion. This is seen with the *Necator americanus* secretory protein (Na-ASP1), which shows potential as a vaccine protein for hookworm infections. Increasing the Na-ASP1 gene copy number caused saturation of secretory capacity in *P. pastoris*, a species of methylotrophic yeast, led to a decreased amount of secreted protein. This was remedied by the overexpression of the protein disulfide isomerase, which allowed for the increased secretion of Na-ASP1 protein in high copy clones (Inan et al., 2006). Another study showed that deletion of obstructive protease genes involved in fission could lead to the enhanced secretion of protease-sensitive human growth hormones (hGH) in *Schizosaccharomyces pombe*. The production of hGH was hampered by the intracellular retention of secretory hGH, and it was determined that the multi-protease deletant strain plays a role in hGH retention. Deletion of *vps10*, which encodes a carboxypeptidase Y sorting receptor and is involved in the traffic between the late-Golgi and prevacuolar compartments, resulted in an approximate twofold increase in hGH secretion (Idiris et al., 2006).

### Combinatorial Biosynthesis

Combinatorial biosynthesis is one genetic engineering application that can modify biosynthetic pathways in order to yield new and altered natural product structures (Hopwood et al., 1985). This approach exploits indiscriminate substrates and uses engineered enzymes and pathways for the production of new natural product analogs.

Modular megasynthases, such as PKS and NRPS enzymes, constitute a class of multifunctional proteins that govern complex enzymatic mechanisms and catalyze multiple reactions useful for combinatorial biosynthesis. Type 1 PKSs consist of multiple modules which are responsible for incorporating acyl-CoAs into a polyketide backbone for elongation. Meanwhile, NRPSs are composed of a modular set of repeating enzyme domains for the activation and incorporation of amino acids (Park and Yoon, 2015). The modular NRPSs typically consist of a condensation domain, adenylation domain, and a thiolation domain, while type I PKSs generally contain a ketosynthase domain, acyltransferase domain, and an acyl carrier protein (Komaki et al., 2015; Skiba et al., 2018). Natural product structures can be modified by mixing and matching the megasynthases at the subunit, module, and domain levels. Genetic manipulation of PKS and NRPS encoding genes can result in predictable changes in structure that is difficult to achieve with standard chemical derivatization or total synthesis methods (Park et al., 2010). This approach to manipulating substrate incorporation and biosynthetic PKS and NRPS machinery has allowed for the generation of a great number of natural product analogs. Examples include erythromycin (McDaniel et al., 1999), pikromycin from type I modular PKS (Yoon et al., 2002) and daptomycin from NRPS (Robbel and Marahiel, 2010).

Post-assembly modifications, such as glycosylation, oxidation, and halogenation are performed by diverse enzymes and can lead to structurally and biologically diverse natural compounds (Park et al., 2010). Sugar moieties attached to the core structure of polyketides or non-ribosomal peptides by glycosyltransferases can also contribute to an extension of combinatorial biosynthesis. Since several glycosyltransferases have been known to be flexible toward sugar donors and sugar accepters, arrays of analogs differing in glycosylation patterns via tailoring enzymes can also be generated by combinatorial engineering of glycosyltransferases from different pathways. For example, one study found that *A. orientalis*-derived glycosyltransferases accepted the unnatural sugar 4-*epi*-vancosamine in the presence of vancomycin pseudoaglycone or the glucosylated teicoplanin scaffold to generate novel hybrid glycopeptide compounds such as 4-*epi*-vancosaminyl form of vancomycin (Losey et al., 2001). Besides sugar biosynthesis, combinatorial biosynthesis can be applied for other modifications such as oxidation and halogenation. Oxidase genes from polyketide pathways have been used to induce structural alterations of important functional groups that are essential for biological activities. It has been reported that 5-*O*-desosaminyl erythronolide A, a potent precursor of ketolides and the latest generation of antibiotic compounds derived from erythromycin A, was produced by expressing the monooxidase gene *pikC* from the pikromycin pathway in a mutant strain of *S. erythraea* lacking of a EryBV glycosyltransferase (Basnet et al., 2008). In addition, a recent study obtained nine analogs of the antitumor antibiotic xantholipin through the individual in-frame mutagenesis of five tailoring enzymes (Zhang et al., 2012). In another study, fluorosalinosporamide, a derivative of the potent anticancer agent salinosporamide A, was produced by replacing the chlorinase gene *salL* from *Salinispora tropica* with the fluorinase gene *flA* from *Salinispora cattleya* (Eustaquio et al., 2010).

However, a common concern with this approach regards limited tolerance of downstream enzymes or domains to the new substance introduced by combinatorial biosynthesis and metabolic engineering. Rational design or directed evolution is one solution to this concern. Rational design is the strategy of creating new molecules with a certain functionality based on predicting how the molecule’s structure will affect its behavior, while directed evolution refers to methods to alter enzyme function using mutagenesis and selection (Nannemann et al., 2011). In a recent study, the reactivity of PikC was modified through protein engineering driven by molecular dynamics and quantum mechanical calculations. The computation-driven PikC engineering yielded a PikC_D50N_ mutant that showed improved catalytic efficiency compared to the wild-type PikC (Narayan et al., 2015). This study demonstrated that a rationally designed protein using a crystal structure of protein and/or a computational analysis can develop a predictive model for substrate scope and selectivity of natural product biosynthesis-mediated reactions. Directed evolution is also a powerful tool to modify the activity of key enzymes responsible for the biosynthesis of natural products and can lead a higher diversity of natural products by generating novel and more potent analogs (Williams et al., 2013). As an example, a few rounds of directed evolution restored and enhanced the activity of an impaired chimerical enterobactin NRPS that has been swapped with a non-cognate aryl-carrier protein (Zhou et al., 2007). In order to reduce the risk of limited tolerance and reduce concerns of efficiency, directed evolution requires a large, high-quality library and an efficient screening strategy. The swapping of functional domains often results in non-functional or heavily impaired chimerical enzymes, and this remains an existing problem when manipulating modular PKS and NRPS systems.

### Mutasynthesis

Novel natural product analogs can also be generated through gene disruption and mutasynthesis. Disruption of a gene, such as a tailoring enzyme acting downstream in a pathway, can serve to introduce a structural change. Two FK506 analogs, 9-deoxo-31-*O*-demethylFK506 and 31-*O*-demethylFK506, were produced by targeting gene disruption in *Streptomyces* sp. MA6548 (Shafiee et al., 1997; Ban et al., 2013). These two recombinant mutants were genetically engineered via disruption of *fkbD* and *fkbM* genes that code for 31-*O*-demethylFK506 methyltransferase and 9-deoxo-31-demethylFK506 hydroxylase/oxidase (Shafiee et al., 1997; Ban et al., 2013). Inactivation of individual domains within the multidomain modular PKSs and NRPSs serves as an alternative to the deletion of a whole gene. Mutasynthesis involves the coupling of a gene inactivation strategy with precursor feeding to generate new structural analogs. Precursor feeding is useful due to the substrate-promiscuity of the biosynthetic enzyme. Precursor feeding may lead to the acceptance of similar substrates or mutasynthons, a natural substrate substitute that can replace the natural substrate of a disrupted gene after being added to the growth medium, to ultimately generate new analogs. Mutasynthesis can generate new analogs for many classes of compounds. For example, the analog cahuitamycin D was produced through mutasynthetic generation with twofold-enhanced biofilm inhibitory activity in comparison to its natural product counterpart (Park et al., 2016). Recently, this approach was applied to generate nonbenzoquinone analogs of the Hsp90 inhibitor geldanamycin, which has anti-proliferative activity on tumor cells (Shin et al., 2008; Wu et al., 2011). By removing the biosynthetic genes for the 3-amino-5-hydroxybenzoic acid starter unit and feeding the culture with various 3-aminobenzoic acids and related heterocycles, a chloro-substituted nonbenzoquinone analog with significantly improved therapeutic properties was produced along with other geldanamycin analogs (Kim et al., 2007, 2009). This has been also seen in the generation of new analogs of rapamycin (Khaw et al., 1998), balhimycin (Weist et al., 2002), and novobiocin/chlorobiocin (Li and Heide, 2005).

## Future Prospects

An increasing number of natural products and natural product-derived compounds have been launched over the years (Butler et al., 2014). Since 2000, 77% of FDA-approved antibiotics are natural products, all of which were derived from microbes (Patridge et al., 2016). There have been extensive reviews of natural products, semi-synthetic natural products, and nature-inspired molecules currently approved by the FDA that show the continued importance of natural products for medicine and health (Sanchez et al., 2012; Newman and Cragg, 2016). Microbial biologics are expected to remain prominent in the global biologics market, which was valued at 277 billion USD in 2015 and was recently estimated to reach 400 billion USD by 2025 (Grand View Research, 2017). While many of the biological activities of microbial natural products and biologics are well known, new advances and insights continue to be discovered. Chemical diversity from microbial natural products continue to be relevant to future drug discovery, with a continuing need for novel drugs with antibiotic, anticancer, and immunosuppressant effects, along with other pharmacological activities (Sanchez et al., 2012).

At the same time, there are a multitude of challenges facing microbial production of natural products and biologics. Some challenges to natural products-based drug discovery involve low production titers, difficulty in product isolation or structural identification. Similarly, there is much room for improvement in terms of the expression of recombinant proteins in microbial platforms. Accumulation of the end product in the microbial cell can cause global stress responses that result in cell growth inhibition. Also, the formation of misfolded and biologically inactive proteins can lower the yield of recombinant proteins. In particular, membrane proteins, high-molecular weight proteins, and multi-domain proteins are often expressed in inclusion bodies. Additionally, expressing eukaryotic proteins in a prokaryotic-based heterologous system can result in a product that is not correctly modified by post-translational enzymes, which are often required for functionality (Rosano and Ceccarelli, 2014). However, a wide variety of engineering strategies can be used with the conventional recombinant DNA technologies, including genome editing, ribosome engineering, precursor engineering, mutagenesis, and overexpression of structural genes, making it possible to facilitate the efficient production of natural products and pharmaceuticals in microbial systems.

Current technologies, such as CRISPR/Cas (Clustered Regularly Interspaced Short Palindromic Repeats/CRISPR associated protein), should also be considered as tools for genome editing for additional improvements and to increase production (Gaj et al., 2013). For example, *in vitro* CRISPR-Cas9 cloning with Gibson assembly has provided an alternate strategy for heterologous expression of cryptic BGCs from genetically recalcitrant actinomycetes strains (Wang J.W. et al., 2015; Jiang and Zhu, 2016). Additionally, CRISPR-Cas9 has many prospects in the analyses of biochemical pathways in *Streptomyces* strains due to the development of a pCRISPomyces expression system (Cobb et al., 2015). With genome editing tools, it is also possible that non-model native hosts can be engineered to be heterologous production hosts to become platforms for combinatorial biosynthesis to create synthetic natural products and natural product derivatives. Some intrinsic limitations for non-model microbial hosts can be also improved by this genetic modification. *Corynebacterium glutamicum*, a GRAS organism, has been used for the industrial production of various amino acids for over five decades (Becker et al., 2011). Recently, it also showed promising potential for use as a protein expression system (Kallscheuer et al., 2016). However, this bacterium has intrinsic disadvantages, including a much lower transformation efficiency and lower yields of protein production. The CRISPR/Cas9 system was successfully employed to disrupt four different genes in *C. glutamicum*, opening new possibilities to use non-model strains as improved cell factories for the production of recombinant proteins and natural products (Peng et al., 2017).

Genome mining is another alternative process to discover secondary metabolites and is done by extracting information from genome sequencing (Bachmann et al., 2014; Zhongyue et al., 2018). Evaluating silent cryptic BGCs through genome mining has provided valuable avenues to generate novel molecules. For example, a genome mining strategy combined with bioinformatics predictions was used to isolate the novel natural product orfamide A by feeding a predicted precursor to a culture of *Pseudomonas fluorescens* (Zerikly and Challis, 2009). In a recent study, genome mining-based combinatorial biosynthesis approach also led to the discovery of new members of the leinamycin family of natural products. Leinamycin has been considered a promising anticancer drug lead due to its potent anticancer activities, unique molecular architecture and interesting modes of action. However, no leinamycin analog had been isolated in the past three decades until this study (Pan et al., 2017).

A combination of approaches can also lead to improvements in the field of microbial natural products, such as gene shuffling and ribosome engineering for increased secondary metabolite production. Additionally, the integration of ‘omics’ information has great potential in natural product drug discovery, such as with metabolomics to accurately quantify biochemical changes and metabolic pathways. Advancements in metagenomics has allowed for further understanding of diverse and complex microbial sources, including lakes, rivers, marine environments and extreme conditions, such as sub-seafloor sites and ice cores (Chandra Mohana et al., 2018).

In terms of structural characterization, X-ray crystallography and cryo-electron microscopy are advanced techniques that can allow for structure solving with high precision. Cryo-electron microscopy has been a leading method for evaluating macromolecular structures at near-atom resolution (Shoemaker and Ando, 2018). For example, single particle electron cryo-microscopy has been used to visualize pikromycin PKS module 5 from *S. venezuelae*, which allowed for 3D map construction with resolutions of 7.3–9.5 Å to reveal secondary structures (Dutta et al., 2014). These techniques are just a few among many that should be considered for structural studies on natural product biosynthesis. Advancements in computational strategies have led to the identification of BGCs in genome sequences and predictions of product chemical structures. Sequencing campaigns for natural product discovery should be directed toward samples likely to yield novel natural products along with well-characterized clades, such as actinomycetes, as they are still a resource for natural product discovery and have yet to be fully exhausted. The development of algorithms to mine the ever-increasing amounts of metagenomic data will allow for the potential of genome mining to be realized (Medema and Fischbach, 2015). Finally, developing additional host platforms for high-throughput refactoring and functional expression of pathways has the potential to overcome current limitations in precursor supply, product toxicity, the ability to express very large gene clusters and more (Schmidt-Dannert, 2015).

Overall, microbial natural products and biologics will continue to broaden their diverse and integral role in human life. The potential for recombinant drugs is expanding through the utilization of new protein production platforms and efforts in product improvement. Microbial cells will remain as potent protein factories because of their versatility and cost-effectiveness. Engineering strategies and recombinant DNA technologies will also allow for the increased production of microbial natural products and recombinant proteins despite the many challenges faced. Continued efforts in natural product analog development will provide an avenue for the discovery of compounds with improved biological activities in comparison to their natural counterparts. Current advanced technologies can be utilized to further advance the field of microbial natural products, which remain a steadfast resource for novel compounds in drug discovery.

## Author Contributions

SP and YY designed, directed, and coordinated this project. JP, MY, AF, MM, NM, JW, EK, HC, JR, MCS, SP, and YY made substantial contributions in providing critical feedback and drafting the manuscript. SP and JP reviewed the final manuscript.

## Conflict of Interest Statement

The authors declare that the research was conducted in the absence of any commercial or financial relationships that could be construed as a potential conflict of interest.
